# TNM-Like Classification: A New Proposed Method for Heart Failure Staging

**DOI:** 10.1155/2013/175925

**Published:** 2013-11-24

**Authors:** Francesco Fedele, Maria Chiara Gatto, Alessandra D'Ambrosi, Massimo Mancone

**Affiliations:** Department of Cardiovascular, Respiratory, Nephrology, Anesthesiology and Geriatric Sciences, Sapienza University of Rome, Policlinico Umberto I, Viale del Policlinico 155, 00161 Rome, Italy

## Abstract

Heart Failure (HF) is an acute or chronic syndrome, that causes a lot of damaging effects to every system. The involvement of different systems is variably related to age and others comorbidities. The severity of organ damage is often proportional to the duration of heart failure. The typology of HF and the duration determine which organs will be affected and vice versa the severity of organ damage supplies precious information about prognosis and outcome of patients with heart failure. Moreover, a classification based not only on symptomatic and syndromic typical features of heart failure, but also on functional data of each system, could allow us to apply the most appropriate therapies, to obtain a more accurate prognosis, and to employ necessary and not redundant human and financial resources. With an eye on the TNM staging used in oncology, we drawn up a classification that will consider the different involvement of organs such as lungs, kidneys, and liver in addition to psychological pattern and quality of life in HF patients. For all these reasons, it is our intention to propose a valid and more specific classification available for the clinical staging of HF that takes into account pathophysiological and structural changes that can remark prognosis and management of HF.

## 1. Introduction 

Heart Failure (HF) is an acute or chronic unhealthy condition, characterized by the inability of the heart to warrant its pump function in terms of adequate oxygen supply to the body tissues. An enchanting explanation of the HF's condition was proposed by Neubauer who defined HF like an “engine out of fuel” [1]. Even though the pump's failure is common to both acute and chronic HF, these differ from one another in HF's duration and the kind of organ damage developed. The prevalence of HF is greater in the population over 50 years old and the incidence is directly correlated with age [2]. The incidence of HF is steadily increasing because the ageing of the population, especially in the occidental world. Moreover, hospitalization and domiciliary treatments for HF represent an important health care burden [3]. The incidence of HF is destined to increase substantially over the next decade [4]. Recent updates of AHA/ACC guidelines about HF focus on diagnosis and management of HF in adults [5]. The current methods for clinical assessment of HF subdivide patients with HF or with high risk for HF in four classes or stages. In the first two stages (A and B), the patients are asymptomatic, whereas in the last two stages, they have a clinically manifested HF (stage C) which becomes refractory to therapy (stage D). The current guidelines modulate therapeutic interventions on the basis of this classification. 

In 2006, Senni et al. [6] claimed that the predictor parameters identified by clinical studies and trials may be often unrepresentative of HF in the community. Senni in his study highlights the importance of prognostic stratification in patients with HF and proposes the use of Cardiovascular Medicine Heart Failure (CVM-HF) index as a valuable tool for the prognosis of stable HF.

 The authors focused not only on cardiac parameters but also on comorbidity of patients with HF and developed a model to evaluate the 1-year risk mortality at all stages of the disease. The CVM-HF index includes 13 variables: age, anemia, hypertension, chronic obstructive pulmonary disease, complicated diabetes mellitus, moderate to severe kidney dysfunction, metastatic cancer, absence of *β* blockers therapy, absence of angiotensin-converting enzyme inhibitors/angiotensin receptor-antagonist, NYHA (New York Heart Association) class III/IV, left ventricular ejection fraction ≤20%, severe valvular heart disease, and atrial fibrillation. Depending on the score achieved, patients are considered in the low-, medium-, and high-risk group [6]. 

In 2009, Cygankiewicz et al. on Behalf of the MUSIC investigators, realized a study that proposed the evaluation of dynamic electrocardiographic measures to identify patients at risk of all-cause mortality and cardiac death [7]. 

In the same year, Vazquez et al. proposed the MUSIC Risk Score, a simple method that evaluates a limited number of variables tested with noninvasive methods. In the MUSIC Risk Score, demographic, clinical, echocardiographic, 12-lead ECG, and 24 h Holter monitoring and laboratory variables were taken into account to predict mortality in ambulatory patients with chronic HF [8]. 

Kalogeropoulos et al. in a recent study, tested the Seattle Heart Failure Model (SHFM) in patients with advanced HF. The study showed that this model overestimates survival, particularly in patients with implanted devices. On the contrary, in blacks, SHFM showed underestimation of the risk in patients with advanced HF [9]. 

Another risk score to assess in-hospital mortality in patients with HF was validated by Peterson et al. using American Heart Association Get With the Guidelines-Heart Failure (GWTG-HF) program data. This risk-score was established using variables identified in the multivariate model. The variables used were age, sex, race, atrial arrhythmia, diabetes, chronic obstructive pulmonary disease, peripheral vascular disease, coronary artery disease, cerebrovascular disease, ischemic etiology, depression, ejection fraction, blood urea nitrogen (BUN), sodium, hemoglobin, creatinine, heart rate, and systolic blood pressure [10]. 

More recently, Peterson et al. indicate a paradoxical inverse relationship between treatment and risk in HF. In practice, higher-risk patients are less likely to receive recommended therapy. This “mismatch treatment” is probably due to the fact that the patients with HF at high risk of mortality have more contraindications to drugs resulting in being ineligible to evidence-based therapy.

For all these reasons, one of the unmet needs in the field of HF is a clinical classification that accurately stratifies our patients in order to (a) make a more precise prognostic evaluation and (b) find the more appropriate treatment, not only in term of efficacy, but also in terms of cost-benefits [11]. A classification of this type, should enable the physician to better manage the patient's illness, but mainly the human and material resources available. In fact, a classification that takes into account comorbidities and their severity in patients with heart failure may also allow a different reimbursement of expenses incurred by national health systems and insurance. Only by framing the main organs involved in HF and their relationship, we can choose the most appropriate therapy to be adopted in a wide range of therapeutic tools available. 

In this setting, we propose a staging system for HF similar to the TNM evaluation used in oncology [12]. The evaluation of myocardial damage is necessary, as well as the type of changes that can occur in the myocardial structure and function. This is the first step where “H”—for Heart—may be the analogue of “T” from the aforementioned TNM. The second step in this HF staging is the assessment of lungs involvement. For their functional and anatomical closeness to the heart, they may be indeed considered, continuing the analogy, as a lymph node station. Finally, remembering the etymological meaning of “metastasis,” that in Ancient Greek signified “what is beyond there,” similarly to the concept used in oncology for metastasization, the “Malfunction” of peripheral organs like kidney, liver, brain, and so forth may represent the final step “M.” 

## 2. Materials and Methods 

### 2.1. “H” for Heart

To evaluate the extension of heart impairment, we propose to consider not only systolic and diastolic functions, in terms of ejection fraction and transmitral flow, but also other characteristical anatomo-functional conditions, as previous myocardial infarction and cardiac remodeling (hypertrophy and or dilatation). Previous myocardial infarction and its extension is a fundamental parameter to be considered in this staging process. Echocardiography is a valid tool to be used for the evaluation of the functional consequences of the ischemic-necrotic process. However, more recently, the magnetic resonance imaging has proved to add further information on tissue characterization, and precisely, on transmural extension of the infarct and myocardial viability [13, 14]. Left ventricular hypertrophy, well evaluable by echocardiography, is the consequence of a series of cardiac and vascular changes. Various etiologies, as hypertension or hypertrophic cardiomyopathies, account for left ventricular hypertrophy [15–17]. Left ventricular remodeling is easily evaluable by echocardiography, that allows the estimate of diameters (systolic and diastolic normal values: 50 ± 5 mm and 31 ± 5 mm, resp. [18]) volumes and shapes. The worst condition of heart in HF is represented by biventricular dysfunction that involves both the left and the right ventricles. 

All these aforementioned parameters have a consolidated prognostic value. Matching in different ways these parameters, grading them from less to more severe conditions, we can obtain a staging of cardiac damage during HF (from H_1_ to H_4_: see scheme in Table 1).

### 2.2. “L” for Lung

**Figure 1:**
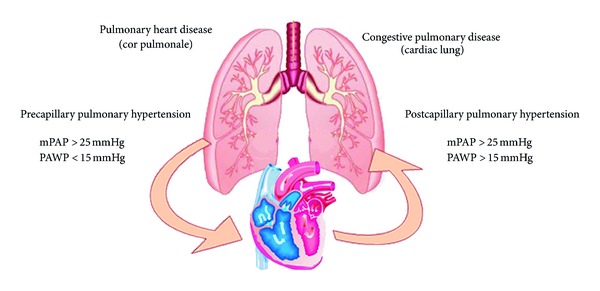
Physiopathology and reference range values in precapillary pulmonary hypertension and postcapillary pulmonary hypertension.

The evaluation of grading and timing of pulmonary system's damage is necessary to give awareness of progress and severity of HF. The clinical manifestations of lungs' implication in HF are multifaceted. In this meaning, the relationship between heart and lungs is to be considered biunique. Even though, in a first moment, left ventricular dysfunction leads to pulmonary congestion, which reveals itself the pulmonary hypertension [19] occurrence, in second time, pulmonary hypertension and pulmonary volume overload precipitate right ventricular dysfunction [20]. Numerous evidences establish that the presence of pressure increment in pulmonary vasculature predicts a poor outcome in patients with HF [21]. Pulmonary hypertension also occurs in patients with HF and preserved ejection fraction [22]. Therefore, considering pulmonary involvement only in patients with HF and impaired ejection fraction would be incorrect [23]. Pulmonary hypertension, as well as HF, is also an age-related disease [24] and is tightly associated with increasing of left atrial diastolic pressures and increasing in systemic vascular resistances. Consequently, to better establish the severity of HF, the functional evaluation of the pulmonary circulation is necessary. Normal pulmonary arterial pressure has been defined with a value of mPAP (mean pulmonary arterial pressure) <25 mm Hg at rest and <30 mm Hg during exercise, while a normal pulmonary arterial wedge pressure (PAWP) has been defined as ≤15 mm Hg. In order to evaluate PAP, echo-Doppler is largely used, provided that the gold standard to evaluate the overall pulmonary hemodynamic is the right cardiac catheterization [25]. An increase in mPAP value > 25 mm Hg with a PAWP value ≤ 15 mm Hg is defined as precapillary pulmonary hypertension with negative influence on right ventricle that can lead to cor pulmonale. On the contrary, mPAP > 25 mm Hg and PAWP > 15 mm Hg, define a condition known as postcapillary pulmonary hypertension and it is characteristic of congestive pulmonary disease (Figure 1).

About congestive pulmonary disease, Gheorghiade [26] purposes a fine differentiation between hemodynamic congestion and clinical congestion, endorsing that the first one is a state of volume overload resulting in augmented left ventricular filling pressures, that precedes cardiopulmonary congestion by several days. Clinical congestion, is the combination of cardiopulmonary and systemic signs and symptoms that result from increased left ventricular filling pressures. The congestive state of pulmonary system occurs in HF when the mPAP and the PAWP are increased. It is also possible that this precarious condition lead to acute HF syndrome with pulmonary edema [27]. 

The early finding of “pulmonary involvement” and their treatment can reduce the progression towards HF. Gheorghiade still supports the usefulness of physical examination to evaluate hemodynamic congestion in the absence of cardiac catheterization. The physical bedside examination includes Valsalva maneuver [28], assessments of orthostatic blood pressure changes, and the heart rate and blood pressure response to sublingual nitroglycerin, in order to identify patients with high left ventricle filling pressures in the absence of signs and symptoms of clinical congestion. Moreover, about lung congestion, also pleural effusion should be considered in decompensated HF [29, 30]. In Table 2(a), we purpose the parameters that can supply a graduation of pulmonary involvement and in Table 2(b) is shown the Lung Staging in HF.

Obviously, a patient with pulmonary edema will be classified as Clinical Congestion L_2_. The Cardiac Lung represents the arterialization of precapillary and postcapillary pulmonary vasculature and it is easy to imagine how this condition maximally compromises the respiratory function. In order to offer the best therapy for each patient, pulmonary parameters (L_*n*_) have to be tightly considered in association with the estimation of cardiac damage expressed by H_*n*_. For example, in treating a patient with pulmonary edema, L_2_ would assume a different role if associated to a cardiac damage estimated as H_1_ or H_4_. In fact pulmonary edema could occur in patients with a preserved left ventricular systolic function, but also in patients with severe systolic disfunction (cardiogenic shock) and left ventricular remodeling (H_4_L_2_) or cardiogenic shock, two different conditions with different therapeutic approaches.

### 2.3. Malfunction of Other Organs

**Figure 2:**
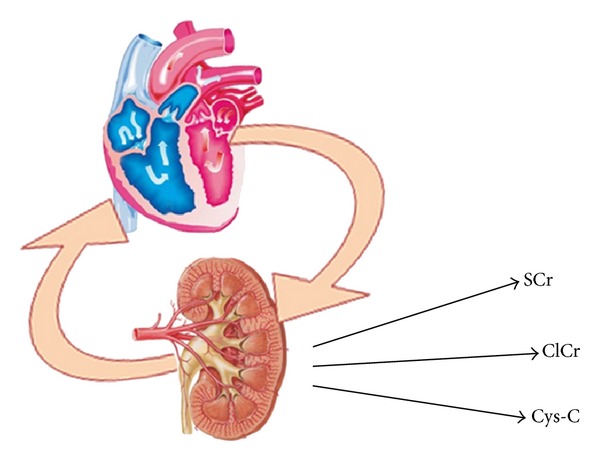
The concept of cardiorenal syndrome that often brings the insaturation of a vicious circle is graphically explicated. SCr: Serum Creatinine; ClCr: Clearance of Creatinine; Cys-C: Cystatine-C.

HF brings countless peripheral systemic signs and symptoms and affects practically all the organs. First of all, there is a correlation between renal and cardiac function. Moreover, the incidence of chronic renal failure is increasing as well as that of HF [31]. Cardiorenal syndromes (CRS) indicate a pathological condition in which there is a tight relationship between cardiovascular system and renal function [32]. CRS is subclassified in five typologies depending on directional relation between kidney and heart. In the type one, a rapid worsening of cardiac function influences the renal function bringing an acute kidney injury (e.g., an acute HF could lead to a cardiogenic renal failure). CRS type two does not result in acute heart damage, but in chronically abnormal heart function that chronically affects the renal function, (e.g., patients with chronic HF often show chronic renal failure). CRS type three is characterized by a sudden worsening of the renal function that leads to acute cardiac injury. CRS type four is represented by a chronic primary renal disease that causes a chronic heart damage overtime. Finally, the CRS type five is characterized by a conjuncted cardiac and renal dysfunctions due to a chronic systemic disease.  Figure 2 shows the graphical representation of cardio-renal syndrome and how the two organs can influence one another. 

Renal function is an important parameter to consider in order to have an overall assessment of patient with HF. It is possible to investigate some parameters in order to define renal function and its impairment grade. 

Creatinine (0.7 to 1.3 VN mg/dL) alone is not a reliable parameter for the measurement of renal function. Indeed, as produced by the muscles and eliminated by the kidney, it increases with muscle mass. Therefore, a value of 1.2 mg/dL is a normal expression of renal function in a patient with muscle mass particularly developed; on the contrary, the same value can “mask” a frank renal failure in a patient with low muscle mass. Furthermore, especially in the early stages of renal failure, small increases in the serum creatinine value indicate significant decreases in glomerular filtration rate (GFR). For these reasons, assessment of renal function cannot only be based on serum creatinine, but it is necessary to determine the GFR: the lower the creatinine clearance is, the lower the patients prognosis is [33]. In this context the evaluation of renal function, by calculating creatinine clearance [34], provides an optimal parameter to estimate peripheral signs of HF. The two main formulas for the calculation of GFR are the Cockroft-Gault and MDRD (Modification of Diet in Renal Disease), although currently the most widely used is a modification of the simplified MDRD formula (sMDRD or simplified MDRD). 

Creatinine clearance, calculated by Cockroft-Gault formula, takes in consideration creatinine serum concentration (mg/dL), age (in years), weight (in Kg), and gender (multiplying the total score for 0.85 in female patients). The complete formula is [(140 − age) × weight]/72 × serum creatinine concentration; the value resulted is multiplied by 0.85 in female patients. The reference values are 57–115 in women and 95–145 in men [35]. 

Simplified MDRD formula, to calculate creatinine clearance, takes in consideration parameters as serum creatinine, ethnicity, and gender. The formula is GFR (mL/min/1.73 m^2^) = 186 × serum creatinine − 1.154 (*μ*mol/L) × age − 0.203 (×0.742 if the patient is a woman) (×1.21 if the patient is black) [36]. Moreover, cystatin C (Cys-c), an endogenous marker of glomerular filtration rate (GFR), is another useful parameter to assess renal function also in patients with HF [37]. Cys-C is a part of the cysteine protease and its serum concentration increases when the GFR diminishes. Since creatinine concentration is influenced by muscle mass and growth and also by other pathological conditions as liver cirrhosis, anorexia nervosa, and so on, Cys-C has proved to be a more reliable parameter in assessing renal function [38]. The usefulness of Cys-C in HF has recently been confirmed by the study of Campbell et al. that demonstrated a poor outcome in patients with high level of Cys-C and impaired renal function [39]. 

It is possible to evaluate blood urea nitrogen (BUN) in order to complete the renal assessment, but it is necessary to consider that BUN is an ambiguous marker of renal impairment because it is strongly influenced by catabolism and catabolic alteration. The parameters to be considered for the evaluation of renal function are shown in Table 3(a).

In order to overview other possible damaged organs in HF, we report an interesting study that supports this concept: “heart disease affecting the liver and liver disease affecting the heart” [40]. HF brings liver complications like alterations of functional liver tests that recover to normal values with the compensation of HF. The study of Allen et al. brings forth that an increment in total bilirubin, alkaline phosphatase, and ALT (alanine transaminase), and a reduction in albumin, are reliable in patients with HF [41]. Moreover, the study proves that this kind of data variably influences the prognosis of patients with HF. To assess liver dysfunction some scores were structured, in particular Child-Pugh score and MELD (Model for End Stage Liver Disease) score. MELD score takes into account total bilirubin, serum creatinine, and INR; Child-Pugh evaluates total bilirubin, serum albumin, INR, ascites, and hepatic encephalopathy. Notice how in both scores created to evaluate liver function, INR and total bilirubin are considered [42]. The INR should be considered carefully since about 1/3 of HF patients is on anticoagulant therapy. 

**Figure 3:**
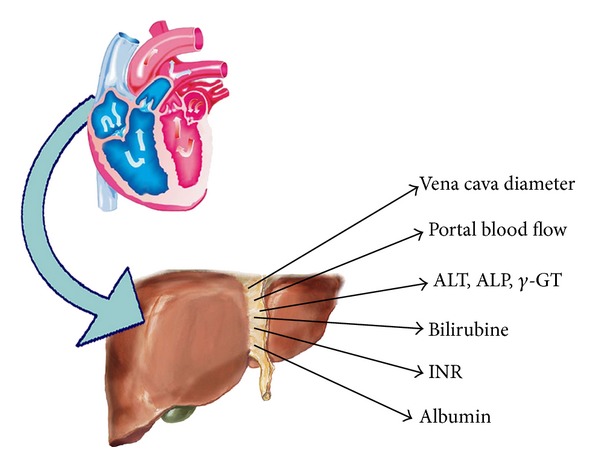
Shows the way of liver's impairment in HF. ALT: Alanine Transaminase; ALP: Alkaline Phosphatase; INR: International Normalized Ratio.

The etiology of liver congestion in HF depends on different variables [43]. When the pulmonary vascular resistance increases and the mPAP is over 25 mm Hg, the repercussion of high pressure induces a volume overload in portal circulation. It is well known that right HF causes liver congestion. For this clinical evidence, in staging HF, it is necessary to consider the hemodynamic and organic function of liver. To obtain an estimate of hemodynamic changes in hepatic district, we can measure the main portal vein flow velocity using duplex sonography [44, 45]. A reduced portal flow that can be detected is likely a consequence of HF. The increment of portal pressure due to right HF could remain silent for years before clinical manifestations occurrence. Another very important parameter that physicians may consider to estimate the congestion state of venous system is the collapse of the inferior vena cava measured with ultrasonography during maximum inspiration. In this regard, a study published by Blehar and coworkers, shows how the diameter variations of the inferior vena cava are related to volume overload in patients with HF [46]. Analogous to the events that occur in kidneys, what occurs in liver during HF (“liver impairment”) is to be considered as a consequence of a more advanced disease. We must underline that, to give a careful estimate of liver damage in HF, it is necessary to evaluate all the parameters summarized in Figure 3 and Table 3(b). In fact, the staging of liver damage based on only one of these parameters is simplistic and misleading because it is known that liver function tests are aspecific indices of deterioration of liver and are therefore to be interpreted in order to make a differential diagnosis with other hepatic diseases. During the investigation of liver's impairment, we can face other clinical features as ascites. Cardiac ascites might indeed develop in patients with a right ventricular failure and systemic venous hypertension [47]. 

Finally, another organ that can be affected by HF is also central nervous system. In fact cerebral hypoperfusion and impaired cerebral function occur in HF [48, 49]. Cerebral vasoreactivity depends on cardiac functional variables. Cerebral complication that can occur in HF is also another consequence of progression of cardiac disease. Other important aspects of brain involvement in HF are psychological troubles. We mainly refer to the depression. In this regard, the research reported by Gottlieb et al. is very interesting [50]. This study demonstrated that a great part of enrolled patients with HF showed signs of depression and that the coexisting depression severely impacts on life's quality and prognosis. Finally, in a comprehensive assessment of the patient with HF it is important to remember a parameter that gives us an idea of the cachectic state of the subject and is representative of multiple organ failure. The most robust and studied marker that can do this is the BMI (mass (Kg)/height (m)^2^) [51, 52]. 

In conclusion, we would like to purpose this score to weight organ's malfunction that can occur in HF. Considering the presence and the number of malfunctioning organs in HF, we can have the M staging as represented in Table 4. The staging of HF influences the prognosis of patients more than their therapy, but, without any doubt, it is necessary to take into consideration any single “malfunction of other organs” in order to guarantee the best treatments and the best quality of life in patients with HF.

## 3. Conclusion 

In our opinion it can be very useful to gain a classification of HF and its progression with a methodological scoring like the TNM evaluation used in oncology. The latest guidelines are mostly based on syndromic and symptomatic classification of HF that, in some cases, could be insufficient. Moreover, the NYHA classification has already been shown to be insubstantial in the evaluation of some kind of patients [53, 54]. We support this viewpoint because this symptomatic classification cannot give a real overview of general health state of the patients with HF. The progression of clinical research in the ambit of HF's classification is still open. What has been written in these pages is intended to provide a classification to proportion our interventions depending on the type of patients we face and to better consider their degree of disease. 

With a TNM-like evaluation we do not risk to rely on single parameters, sometimes aspecific, but we have the opportunity to evaluate all the necessary parameters and enclose them in a single compressive classification representing the patient in that moment. This classification will allow us to use a traditional therapy for patients in initial stage of HF (H_1_L_1_M_0_) and to apply second-tier line therapies in patients with H_2_L_2_M_*n*_ stage. In fact, the presence of malfunction of other organs authorizes the physicians to employ type of therapies that will be not only cardioprotective, but also nephroprotective, hepatoprotective, and so on, justifying the increased cost of therapy in proportion to the benefits obtained. Besides, this type of classification can be very useful in assessing the true end-stage patients (H_4_L_3_M_3_). In these cases, the classification HLM allows us to pursue the most appropriate therapy targeting the quality of life remained with palliative support. 

Starting with a patients' followup, applying this new staging system, the future prospect is to stratify prognosis, consider the course of therapy, and evaluate cardiovascular events of patients affected by HF.

## Figures and Tables

**Table:** (a)

Cardiac damages	
Hypertrophy	
Transmitral flow	
Previous N-STEMI	
Previous STEMI	
Ventricular remodeling	
Ejection fraction < 35%	

**Table:** (b)

Staging	
H_1_: impaired systolic or diastolic function of LV without structural damage	
H_2_: LV with systolic or diastolic dysfunction and structural damage (hypertrophy previous myocardial infarction)	
H_3_: systolic and diastolic dysfunction (and/or EF < 35%) with left ventricular remodeling	
H_4_: biventricular systolic and diastolic dysfunction	

**Table:** (a)

Parameters of pulmonary damage	
Precapillary pulmonary hypertension (mPAP > 25 mmHg PAWP < 15 mmHg)	
Postcapillary pulmonary hypertension (mPAP > 25 mmHg PAWP > 15 mmHg)	
Pleural effusion	
Pulmonary edema	

**Table:** (b)

	Staging
L_1_	Hemodynamic congestion
L_2_	Clinical congestion
L_3_	Cardiac lung

**Table:** (a)

Parameters of kidney damage	
Glomerular filtration rate	
Blood urea nitrogen	
Serum creatinine	
Clearance of creatinine	
Cystatin C	

**Table:** (b)

Parameters of hepatic damage	
*γ*-GT	
Bilirubine (total) increased	
ALT, AST	
Alkaline phosphatase increased	
Albumin decreased	
Diminished INR value	
Ascites	
Impairment of portal blood flow	
No changes of inferior vena cava diameter variation during inspiration	

**Table 4:** Staging of malfunction of other organs.

Score	Staging
0	M_0_: no malfunction of other organs
1	M_1_: single organ damage due to HF
2	M_2_: double organ damage due to HF
≥3	M_3_: multiple organ damage
